# CCR2^+^ monocytes/macrophages drive steroid hormone imbalance-related prostatic fibrosis

**DOI:** 10.1038/s41598-024-65574-4

**Published:** 2024-07-08

**Authors:** Petra Popovics, Samara V. Silver, Kristen S. Uchtmann, Lisa M. Arendt, Chad M. Vezina, William A. Ricke

**Affiliations:** 1 Department of Microbiology and Molecular Cell Biology, Eastern Virginia Medical School, Norfolk, VA 23507 USA; 2 The Leroy T. Canoles Jr. Cancer Research Center, Eastern Virginia Medical School, Norfolk, VA 23501 USA; 3 Department of Urology, School of Medicine and Public Health, University of Wisconsin-Madison, Madison, WI 53705 USA; 4 George M. O’Brien Center of Research Excellence, School of Medicine and Public Health, University of Wisconsin-Madison, Madison, WI 53705 USA; 5 Department of Comparative Biosciences, School of Veterinary Medicine, University of Wisconsin-Madison, Madison, WI 53706 USA

**Keywords:** Prostatic fibrosis, Macrophages, Extracellular matrix, Benign prostatic hyperplasia, Lower urinary tract symptoms, Cell biology, Prostate

## Abstract

Benign Prostatic Hyperplasia (BPH) is a complex condition leading to Lower Urinary Tract Symptoms in aging men, characterized by cellular proliferation, smooth muscle dysfunction, inflammation, and fibrosis. While BPH is known to involve heightened macrophage infiltration, the specific contribution of infiltrating monocytes/macrophages to the disease mechanism remains uncertain. This research explores the impact of reducing circulating monocytes and subsequently limiting their tissue infiltration by using *Ccr*2 knockout (*Ccr*2-KO) mice. *Ccr*2-KO and wild type mice were implanted with testosterone and estradiol (T + E2, 25 mg + 2.5 mg) pellets. Urinary function was assessed via weekly void spot assays over 12 weeks, and prostatic macrophage levels were visualized and quantified in tissue sections using an F4/80 antibody. Additionally, Ki-67 staining was used to evaluate cell proliferation, and picrosirius red staining to assess collagen accumulation. Increased voiding frequency which developed in T + E2 mice, was significantly ameliorated in *Ccr*2-KO mice, however, both *Ccr*2-KO and wild type (WT) mice showed increased bladder weights after three month, representing a hypertrophic response to bladder outlet obstruction. T + E2 substantially increased the density of macrophages in WT but not *Ccr*2-KO mouse prostate. Proliferation rate, as indicated by Ki-67 positivity, was elevated in the vental and anterior prostate lobes but was only marginally reduced in *Ccr*2-KO mice. Most importantly, a significant prostatic collagen accumulation was observed in WT mice that was markedly reduced by *Ccr*2 deficiency post T + E2 treatment. The absence of *Ccr*2 mitigates urinary dysfunction and alters prostatic macrophage levels and collagen accumulation in steroid hormone imbalance. These findings suggest a crucial role for monocyte infiltration, giving rise to macrophages or other cell derivatives, to drive fibrosis.

## Introduction

Benign Prostatic Hyperplasia (BPH), characterized by non-malignant tissue growth, along with inflammation, smooth muscle dysfunction, and fibrosis, is a key pathology underlying Lower Urinary Tract Symptoms (LUTS) in aging men^1–3^. LUTS, which can include nocturia, incomplete emptying, and weak stream, often result from these benign prostatic changes. The prevalence of BPH in the sixth decade of life is 70%, with moderate-to-severe LUTS impacting 33% of men in their 60s, significantly impairing their quality of life^4^. Current medical interventions predominantly address smooth muscle dysfunction and cellular proliferation, leaving inflammation and fibrosis in the prostate inadequately treated.

Constant remodeling of the prostatic immune environment seems to be a key factor in BPH pathogenesis. In LUTS patients, an increased prostate size is associated with a higher number of immune cells^5^ with T-lymphocytes and macrophages being the predominant cell types involved^6^. Macrophage numbers are also significantly increased in BPH tissues compared to normal prostates, underscoring their vital role in the pathogenesis of BPH^7^. In murine models, the insertion of testosterone and estradiol pellets (T + E2 model) mimics the imbalance in steroid hormone levels observed with aging, encapsulating the complete pathological spectrum of BPH and associated lower urinary tract dysfunction^8,9^. Our prior research in this model has highlighted the predominance of macrophages and identified the disease-specific emergence of luminal foam cells, which are lipid-laden macrophages^9^. However, it remains unclear whether the infiltration of macrophages is a result of the disease process or a contributing factor to BPH progression. Notably, macrophages have been shown to stimulate the proliferation of prostate stromal and epithelial cells in vitro^7,10^ raising questions about the preservation of these roles during disease pathogenesis.

Two main monocyte subsets are found within the circulation. In mice, CCR2^high^ monocytes represent the classical type that can differentiate into macrophages or dendritic cells in the infiltrated tissue and carry antigens to lymph nodes. In contrast, CX3CR1^high^ non-classical monocytes are early responders to pro-inflammatory signals^11^. CCR2, along with its associated ligands, namely chemokine [C–C motif] ligand 2 (CCL2), CCL7, CCL8, CCL12, CCL13, and CCL16, are essential for the exit of classical monocytes from the bone marrow^12–14^. In addition, CCR2 also mediates the infiltration of monocytes at inflammatory sites^15^. This results in a significantly reduced number of basal and inflammation-induced circulating classical, and even non-classical monocytes in *Ccr*2 knockout (*Ccr*2-KO) mice^16,17^. Consequently, *Ccr*2-KO mice are often utilized to study the role of infiltrating monocytes in disease models.

In this study, we generated the T + E2-induced BPH model in *Ccr*2-KO and wild type (WT) mice, tracked changes in urinary frequency for 12 weeks and analyzed pathological changes across all mouse prostate lobes. We identified that monocyte infiltration is essential in the development of fibrosis, but, surprisingly, has little effect on proliferation. This is the first study identifying the pivotal role of infiltrating classical monocytes as drivers of prostatic fibrosis.

## Materials and methods

### Mice and surgery

All experiments were conducted under approved protocols from the University of Wisconsin (ID: M005570-R01-A04) and in accordance with the National Institutes of Health Guide for the Care and Use of Laboratory Animals. In addition, the authors complied with the ARRIVE guidelines. B6.129(Cg)-*Ccr2*^tm2.1lfc^/J (*Ccr2*-KO) were obtained from The Jackson Laboratory and were bred at the UW-Madison Biomedical Research Model Services. C57BL/6 J were used as controls, as suggested by The Jackson Laboratory. Animals were maintained on a strict 12:12 h light–dark cycle in a temperature- and humidity-controlled facility with water and food provided ad libitum. Testosterone (25 mg) and estradiol mixed with cholesterol (2.5 mg and 22.5 mg, respectively) were compressed as pellets and were surgically implanted subcutaneously as previously described^8^. Mice that underwent sham surgery were used as controls. Tissues were harvested twelve weeks after surgery. The first experiment was conducted using 4–5 mice/group to assess urinary frequency and bladder weights. Another experiment was performed to add 5 C57BL/6 J and *Ccr2*-KO mice to the respective T + E2 groups for histological analyses.

### VSA

Void spot assays (VSAs) were executed as previously described^18,19^. Briefly, mice were isolated individually in cages lined with filter paper for a duration of four hours, during which they had access to food but were deprived of water. Filter papers were then dried and imaged using an Autochemi AC1 Darkroom ultraviolet imaging system (UVP, Upland, CA, USA). The quantification of void spots was performed using the Void Whizzard software^20^. To acclimate the mice to the VSA environment, an initial assay was conducted without data analysis, serving to establish baseline VSA parameters in subsequent experiments. This assay protocol was consistently replicated on a weekly basis over a period of 12 weeks.

### Immunohistochemistry and picrosirius red (PSR) staining

Tissue sections were de-paraffinized and rehydrated. Antigen retrieval was performed using a decloaking chamber (Biocare Medical, Pacheco, CA, USA) and employing citrate buffer at pH 6.0. To inhibit the activity of endogenous peroxidases and reduce non-specific binding, Bloxall (Vector Laboratories, Burlingame, CA, USA) and horse serum (10%) were applied. The sections were then treated with primary antibodies: F4/80 (1:500 dilution, MA5-16,363, Invitrogen, Waltham, MA, USA) and Ki-67 (1:3500 dilution, AB9260, Abcam, Waltham, MA, USA) which were incubated overnight. This was followed by a 30-min incubation with an HRP-conjugated horse anti-rabbit IgG Polymer (Vector Laboratories) as the secondary antibody. The detection signal was developed using the SignalStain DAB Substrate Kit (Cell Signaling). On separate tissue sections, fibrous collagens were stained using the PSR Kit (Polysciences, Warrington, PA) according to the manufacturer`s protocol. All four experimental groups were represented on each tissue sections and all sections were processed together for a particular experiment.

### Imaging and analysis

Tissues were imaged with a Mantra 2 Quantitative Pathology Workstation (Akoya Biosciences, Marlborough, MA, USA) with a 40 × objective. Six representative images were taken per tissue. Area not containing tissue and the luminal space were removed to acquire the total tissue area for normalization. Number of positive cells for Ki-67 and F4/80 were counted manually. Cell counts were normalized to tissue area. PSR fluorescent counts were detected through the Alexa 594 filter on the Mantra Workstation and were normalized to the tissue area. Total intensity counts were used that are the sum of intensity of each pixel thereby incorporating both the area and the intensity of collagen staining into the calculation.

## Results

### T + E2-induced urinary dysfunction is ameliorated in Ccr2-KO mice

**Figure 1:**
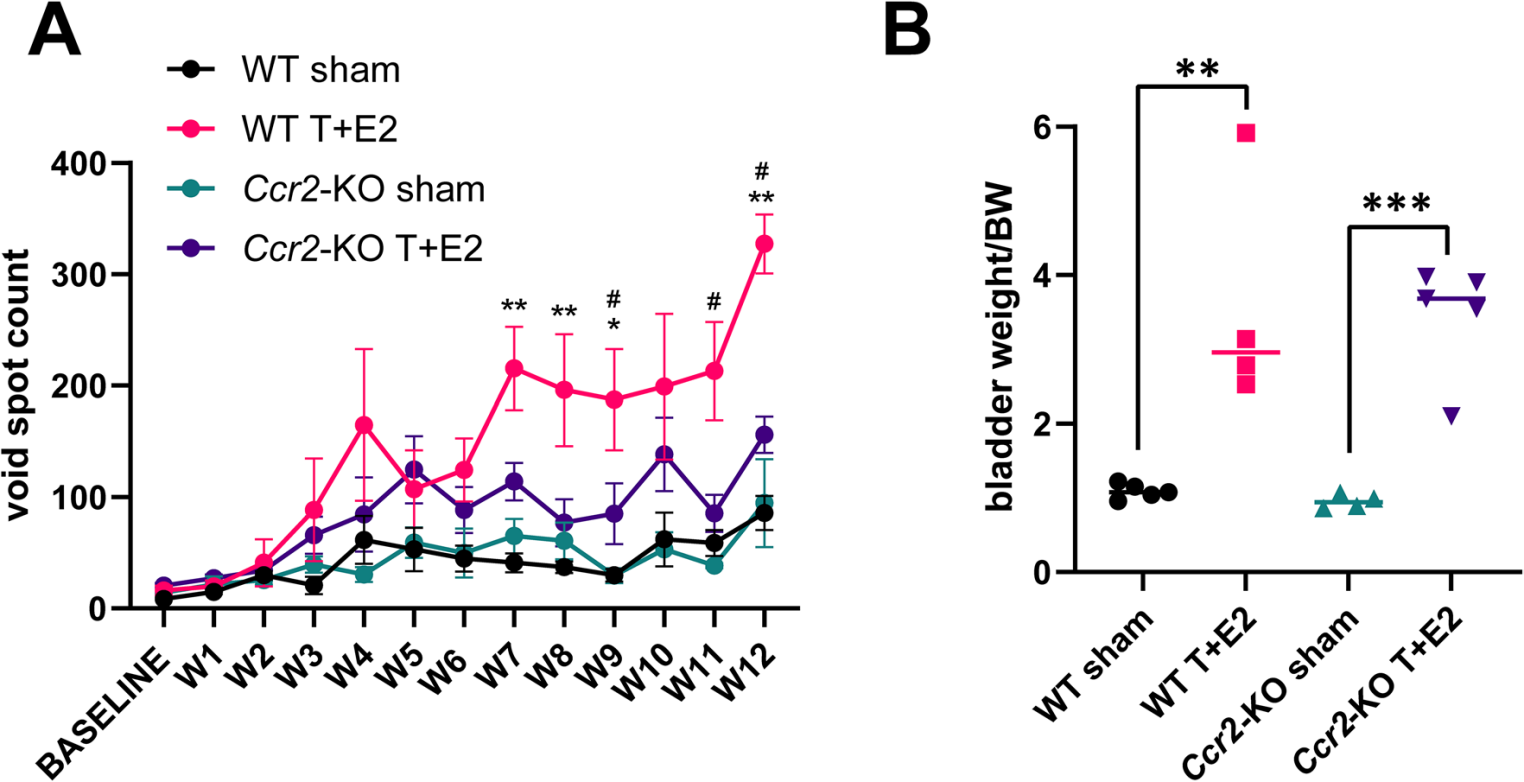
CCR2 deficiency suppresses urinary frequency but not bladder weight in mice implanted with testosterone and estradiol pellets (T + E2). Panel (**A**) shows that significantly increased void spots were detected on weeks 7, 8, 9, 11 and 12 in wild type (WT) mice vs. sham controls, but not in *Ccr2*-knockout (*Ccr2*-KO) mice. Significance was assessed by Kruskal–Wallis test for each week. Panel (**B**) shows bladder weights normalized to body weight (BW) three months after pellet implantation for each group. Significance was assessed by Student’s t-test. *: WT sham versus WT T + E2, #: *Ccr2*-KO sham versus WT T + E2. */#: *p* < 0.05, **: *p* < 0.01, ***: *p* < 0.001. WT T + E2 versus WT T + E2 was not significant.

*Ccr*2-KO and wild type male mice were implanted with testosterone and estradiol (T + E2) pellets to induce steroid hormone imbalance. Urinary function was assessed with weekly VSA for 12 weeks. This identified a significant increase in WT mice receiving pellet implantation on weeks seven (5.3-fold), eight (5.3-fold), nine (6.3-fold), eleven (3.6-fold) and twelve (3.8-fold) compared to WT sham controls, whereas no increase was detected in *Ccr*2-KO T + E2 mice (Kruskall-Wallis test) (Fig. 1A). T + E2 significantly increased bladder weights, an indicator of obstruction-induced hypertrophy, in both WT and *Ccr*2-KO mice (Fig. 1B).

### Ccr2-KO mice are resistant to the T + E2 mediated increase in prostatic macrophages

**Figure 2:**
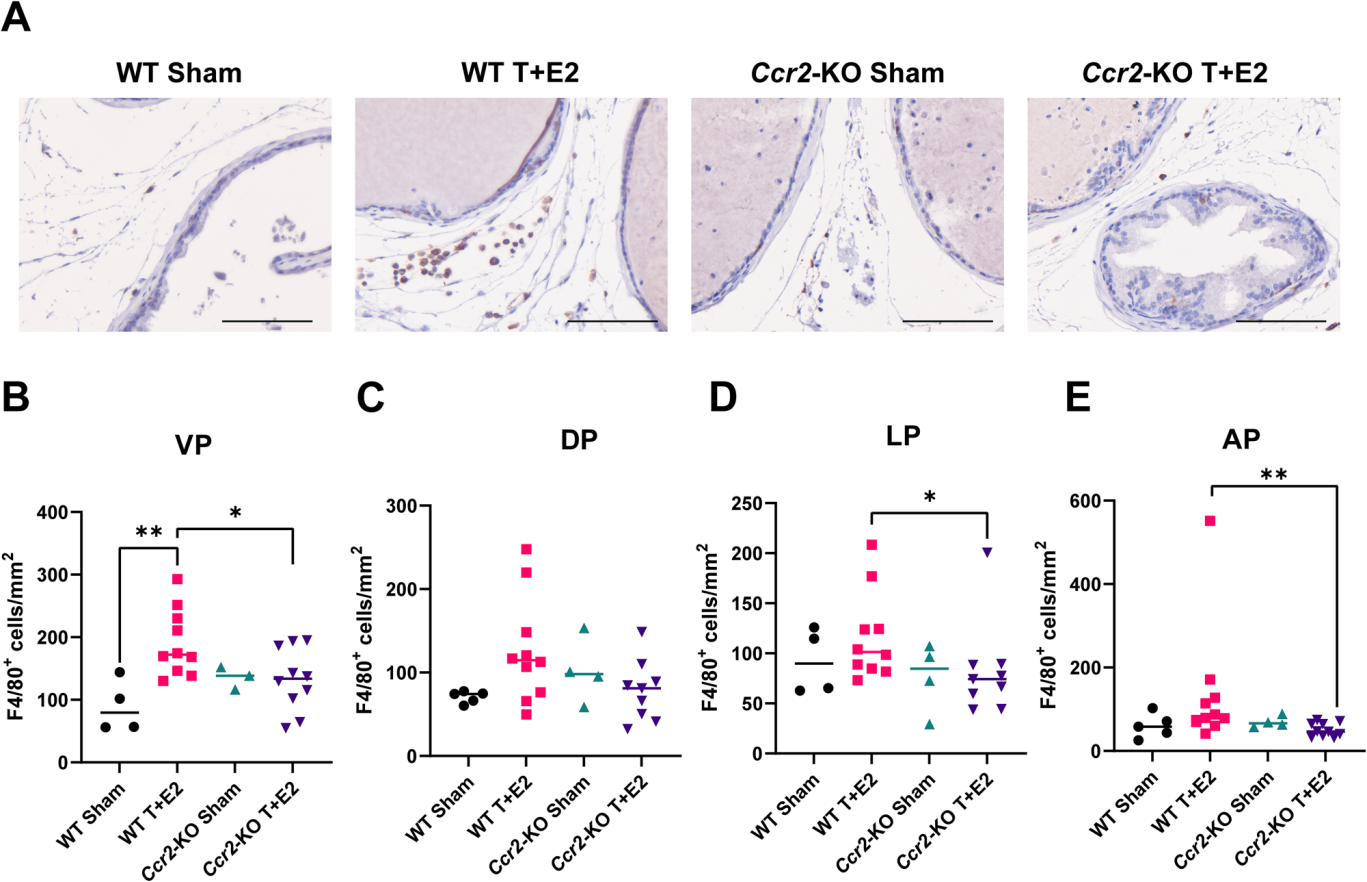
Loss of CCR2 abolishes the steroid hormone imbalance-induced increase in prostatic macrophages. Panel (**A**) contains representative images of immunohistochemistry for the F4/80 macrophage marker on ventral prostate tissue in wild type (WT) and *Ccr2* knockout (*Ccr2*-KO) mice and their respective sham controls. Panels (**B, C, D** and **E**) show the quantification of F4/80^+^ cells normalized to tissue area in mm^2^ in the ventral (VP), dorsal (DP), lateral (LP) and anterior prostate lobes (AP), respectively. Significance was assessed by Mann–Whitney pairwise comparisons. Scale bar represents 100 µm. *: *p* < 0.05, **: *p* < 0.01.

Our previous research demonstrated that implantation of steroid hormones led to a heightened accumulation of prostatic macrophages, predominantly observed in the ventral lobe^9^. In the present investigation, at 12-weeks post T + E2 implantation, we observed a substantial elevation in macrophages, as identified by the F4/80 marker, in the ventral lobe of WT mice compared to WT sham controls (Fig. 2A,B). The T + E2-mediated increase in DP macrophage density was also nearing significance (*p* = 0.0753) in WT mice using the non-parametric Mann- Whitney test, whereas with Welch`s test, for which the variances of the two groups are not assumed to be equal, we were able to determine a significant increase (*p* = 0.0222). Welch`s test is appropriate based on the difference between group sizes (5 vs. 10). Importantly, the number of macrophages in the ventral, lateral, and anterior, but not the dorsal lobe, was significantly lower in *Ccr*2-KO vs. WT mice receiving hormone pellets (Fig. 2B–E).

### T + E2 induced prostate cell proliferation is only marginally affected by *Ccr2*-loss

**Figure 3:**
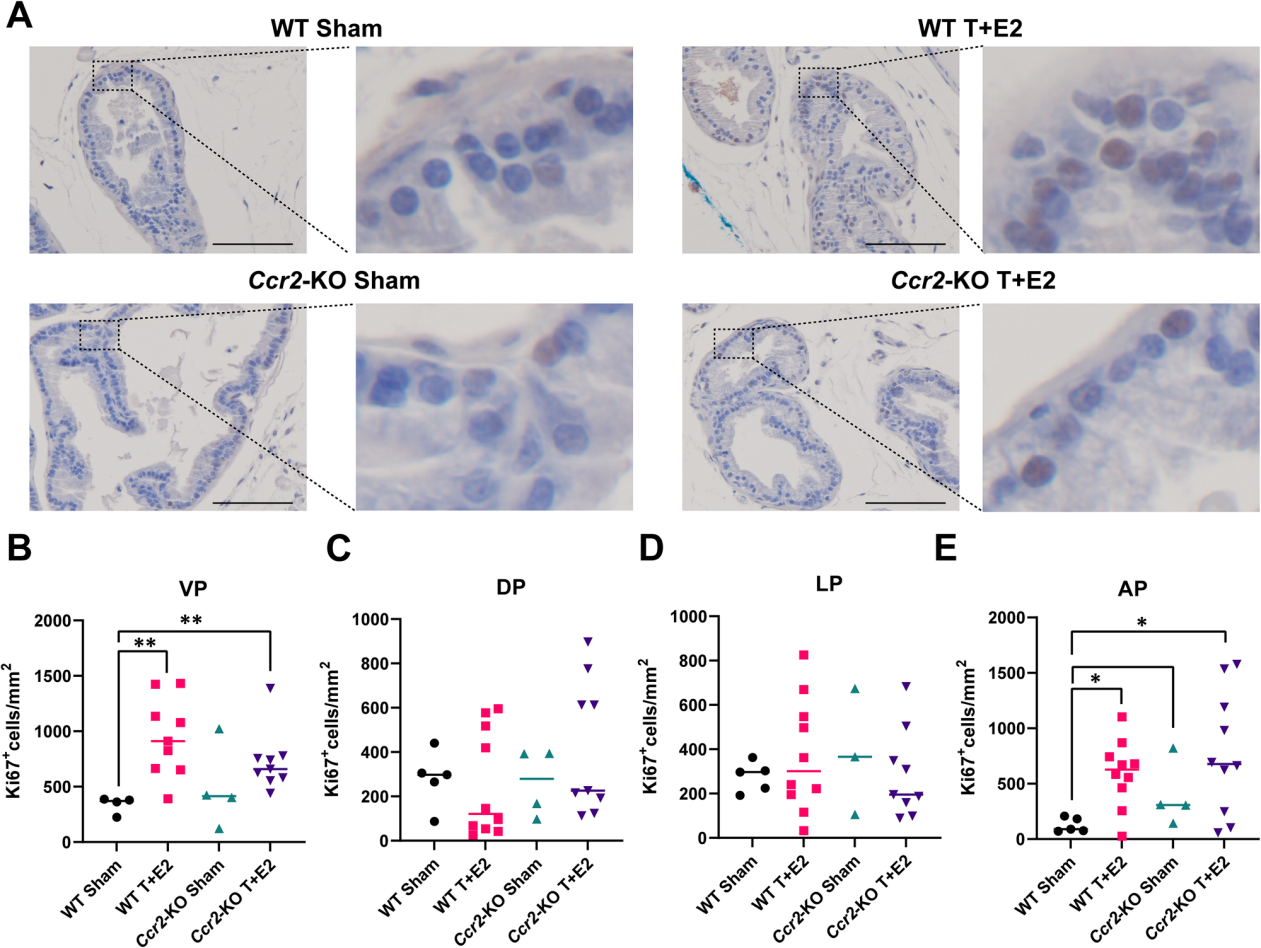
Prostatic proliferation is unaffected by CCR2 deficiency. Panel (**A**) contains representative images of immunohistochemistry for Ki-67 proliferation marker on ventral prostate tissue in wild type (WT) and *Ccr2*-KO sham and testosterone and estradiol (T + E2)-implanted mice. Panels (**B, C, D** and **E**) show the quantification of cells with nuclear Ki-67 positivity normalized to tissue area in mm^2^ in the ventral (VP), dorsal (DP), lateral (LP) and anterior prostate lobes (AP), respectively. Significance was assessed by Mann–Whitney pairwise comparisons. Scale bar represents 100 µm. *: *p* < 0.05, **: *p* < 0.01.

Previous research has demonstrated that the impact of steroid hormone implantation on cell proliferation varies among different lobes of the mouse prostate^9^. In the current study, we observed a notable increase in the number of Ki-67-positive cells, indicating an increase in the number of cells entering the proliferative phase of the cell cycle, in the ventral and anterior prostate lobes, but not in the dorsal and lateral lobes of WT T + E2-treated mice compared to sham controls (Fig. 3A–E). Similarly, *Ccr*-KO mice exhibited an increase in Ki-67-positive cells, which was statistically significant when compared to wild-type sham-treated mice (Fig. 3B,E). The absence of an increase in proliferation in the dorsal and lateral lobes corroborates our previous findings, and suggest that the increase in weight might not be caused by increased cell proliferation in these lobes^9^.

### Perturbed monocyte infiltration halts prostatic collagen accumulation

**Figure 4:**
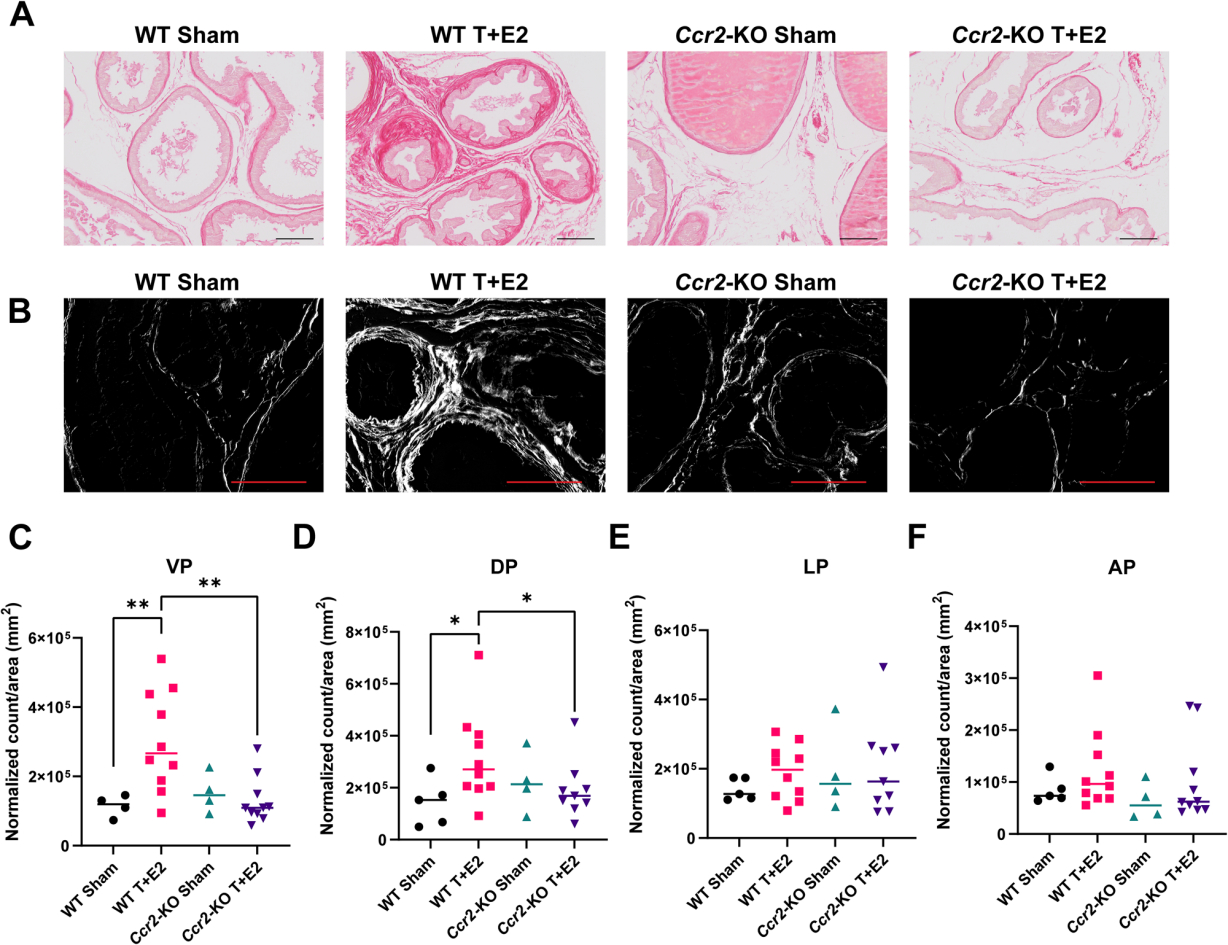
Loss of CCR2 prevents steroid hormone imbalance-induced collagen accumulation. Panel (**A**) contains representative brightfield images of picrosirius red staining of the ventral prostate in wild type (WT) and *Ccr2* knockout (CCR2-KO) mice and their respective sham controls at 20 × magnification. Panel (**B**) shows the fluorescent detection of picrosirius red staining in the same tissues at 40 × magnification. Panels (**C, D, E** and **F**) show the quantification of collagen intensity count normalized to tissue area in mm^2^ in the ventral (VP), dorsal (DP), lateral (LP) and anterior prostate lobes (AP), respectively. Significance was assessed by Mann–Whitney pairwise comparisons. Scale bar represents 100 µm. *: *p* < 0.05, **: *p* < 0.01.

Prostatic fibrosis, characterized by the build-up of fibrillar collagens, emerges as a delayed consequence of steroid hormone implantation over a period of 12 weeks^9^. To enhance the sensitivity of detecting collagen in all lobes of the prostate, we adapted and modified a method from a previous study by Wegner et al., which leveraged the fluorescent characteristics of picrosirius red^21^. Employing this modified approach, we observed a marked increase in the density of fibrillar collagen in both the ventral and dorsal lobes following T + E2 pellet implantation, an effect not seen in the lateral or anterior lobes (Fig. 4A–F). Most notably, the accumulation of collagen induced by T + E2 was significantly reduced by the loss of *Ccr2* (Fig. 4A–D).

## Discussion

Our study utilized a mouse model of BPH that closely represents the pathological aspects of the disease. This model was employed to assess the role of monocyte infiltration in benign prostate pathology. In *Ccr2*-KO mice, where circulating monocyte levels are markedly reduced, we tested the effects of monocyte-derived macrophages on prostatic pathological components and urinary function. In CCR2 deficiency, monocyte migration is compromised, leading to their sequestration in the bone marrow^17^. Although CCR2 plays a major role in neutrophil migration^22^, neutrophil numbers are negligible in the T + E2 model^9^. Additionally, CCR2 has been shown to direct the migration of T-cells^23^, and may slightly affect the number of B-cells^24^, however, these cells also do not undergo significant changes in our steroid hormone imbalance model^9^. This provides us with high confidence that the specific role of monocyte infiltration can be accurately assessed in *Ccr2*-deficient mice.

Mice exposed to T + E2 develop specific urinary patterns, characterized by droplet voiding and an enlarged bladder volume, primarily due to urinary obstruction^8^. This particular voiding phenotype, marked by an increased frequency of urination, was successfully replicated in WT mice in our study. However, it was notably absent in mice with *Ccr*2 deficiency, highlighting a distinct divergence in urinary behaviors depending on monocyte infiltration. Despite this difference in voiding patterns, an interesting observation was that both WT and *Ccr*2-KO mice exhibited a similar increase in bladder weight. This suggests that, although throughout the experiment, urinary frequency was ameliorated by the loss of monocyte infiltration, the level of obstruction had reached a comparable degree in both groups at the 3-month mark. Since the major outcome of *Ccr2* deficiency was the suppression of fibrosis, we can speculate that this pathological feature is required for the maximal urinary dysfunction developing in response to steroid hormone imbalance, however, further studies will be required to confirm the exact contribution of prostate pathologies.

Based on our prior study^9^, we expected and confirmed that the highest increase in macrophages is found within the ventral prostate in response to T + E2 pellet implantation in WT mice. In addition, we identified a moderate increase in the dorsal lobe. In contrast, macrophage populations in *Ccr2*-KO mice remained unaltered upon exposure to T + E2 treatment. While CCR2^High^ monocytes are typically classified as the classical monocyte subset, it is also known that circulating non-classical monocytes are suppressed during inflammatory episodes in *Ccr2*-KO mice^16^. This pattern of response suggests that the deletion of *Ccr2* gene function in these mice leads to a general impediment of monocyte infiltration into tissues that prevented the rise in F4/80^+^ macrophage numbers in the prostate in our study.

While previous research indicates that macrophages facilitate the proliferation of prostate stromal and epithelial cells in vitro, our findings suggest that macrophage infiltration does not influence proliferation (Ki-67 positivity) in the T + E2 model. The discrepancy observed between in vivo and in vitro systems could be attributed to a multitude of factors, contingent upon the specific model employed and the type of macrophage activation. Previous studies have demonstrated that co-culturing bone marrow-derived macrophages with prostate stromal cells can enhance stromal proliferation, a process mediated by the chemokine CCL3^7^. Additionally, the co-culture of macrophages with prostate epithelial cells has been shown to promote epithelial cell proliferation, potentially driven by osteopontin^10^. These findings suggest that tissue macrophages could indeed stimulate the proliferation of resident prostate cells. However, this proliferative effect may be more prominent in models exhibiting a pronounced inflammatory phenotype. Our prior data indicated the coexistence of both pro- and anti-inflammatory macrophage activation within the T + E2 model. However, the lack of significant T-cell and neutrophil accumulation during the early stages of steroid hormone imbalance^9^ indicates that classical acute inflammation does not develop. In addition, a critical limitation of our study is its temporal constraint; data were only captured three months post-implantation of hormone pellets and it is conceivable that cell proliferation might have been affected at different stages within this timeframe.

Recent research has identified prostatic fibrosis as a key pathological component of BPH/LUTS. Studies show that periurethral prostate tissue in LUTS patients is notably stiffer, primarily due to an increase in extracellular matrix components, particularly fibrillar collagens^1,25,26^. This collagen accumulation within the prostate has been observed in mouse models of lower urinary tract dysfunction (LUTD) mimicking conditions such as obesity, aging, prostatic inflammation, and steroid hormone imbalance^9,18,27,28^. A pivotal discovery in our study is the significant reduction of prostatic collagen buildup in both the ventral and dorsal lobes of the prostate when monocyte infiltration is impeded. This finding highlights the essential role of monocyte-derived macrophages in promoting fibrosis. Interestingly, no difference was noted in cellular proliferation, emphasizing the impact of fibrosis on urinary dysfunction observed in this model.

CCR2^+^ monocytes have been recognized as key players in fostering fibrosis in various tissues, including the colon, lung, and kidney^29–31^. In the case of colon fibrosis, these monocytes and their fibrocyte derivatives are instrumental in regulating extracellular matrix dynamics and advancing fibrosis, primarily through the release of the tissue inhibitor of metalloproteinase-1 (TIMP-1)^29^. Similarly, in the lung affected by cystic fibrosis, CCR2^+^ monocytes/macrophages contribute to heightened levels of TGF-β in tissues and an increase in collagen production^30^. While our study did not pinpoint the precise molecular pathways through which monocytes, macrophages, or potentially fibrocytes, promote fibrosis, it underscored the essential role of monocyte infiltration in the broader context of fibrosis development in the prostate.

In summary, our study utilizing a BPH mouse model revealed that *Ccr2* deficiency hinders monocyte infiltration and macrophage increase in the prostate. This resulted in the amelioration of urinary dysfunction in *Ccr2*-KO mice, but a shared increase in bladder weight indicating similar levels of urinary obstruction reached only after three months. Notably, the absence of monocyte infiltration in *Ccr2*-deficient mice led to a significant reduction in prostatic collagen buildup, implicating monocyte-derived macrophages in driving prostatic fibrosis. Future goals should focus on elucidating the molecular mechanisms by which monocytes and macrophages contribute to prostate pathology, especially their role in fibrosis and their impact on urinary dysfunction.

## Data Availability

All data generated during the current study is available from the corresponding authors upon reasonable request.
